# The Impact of the COVID-19 Pandemic on Hernia Surgery: The South-East Scotland Experience

**DOI:** 10.7759/cureus.29532

**Published:** 2022-09-24

**Authors:** Anne S Ewing, Rory McFadyen, Katherine Hodge, Cathleen M Grossart, Barbora East, Andrew C de Beaux

**Affiliations:** 1 Department of General Surgery, Royal Infirmary of Edinburgh, Edinburgh, GBR; 2 Department of Surgery, Motol University Hospital, Prague, CZE

**Keywords:** covid-19 impact on general surgery, covid-19, emergency and elective surgery, inguinal hernia repair, incisional ventral hernia

## Abstract

Aim

The coronavirus disease 2019 (COVID-19) pandemic resulted in a lockdown in South East Scotland on 23 March 2020. This had an impact on the volume of benign elective surgery able to be undertaken. The degree to which this reduced hernia surgery was unknown. The aim of this study was to review the hernia surgery workload in the Lothian region of Scotland and assess the impact of COVID-19 on hernia surgery.

Methods

The Lothian Surgical Audit database was used to identify all elective and emergency hernia operations over a six-month period from 23 March 2020 and for the same time period in 2019. Data were collected on age, gender, anatomical location of the hernia, hernia repair technique, and whether elective or emergency operation. Statistical analysis was performed using the chi-squared test in R-Studio, with a p-value of <0.05 accepted as statistically significant.

Results

The total number of hernia repairs reduced considerably between 2019 and 2020 (570 vs 149). The majority of this can be explained by a decrease in elective operating (488 vs 87), with the percentage of elective repairs reducing significantly from 85.6% to 58.4% (p<0.001). The inguinal hernia subgroup had a 24% rise in emergency operations from 21 to 26 operations, despite a reduction from 270 to 84 total inguinal repairs. There were just two elective hernia repairs carried out in the first three months of the 2020 study period (5.6% of all operations for April-June) compared to 265 (87.7%) for the same period in 2019 (p<0.001). No statistically significant differences were observed in the rates of laparoscopic versus open operating techniques across the two study periods on any analysis. The age and gender of the patients were similar over the two time periods.

Conclusion

The COVID-19 pandemic led to a marked reduction in the number of elective hernia repairs (especially incisional hernia surgery), with the effect most pronounced over the first three months of lockdown. Despite an overall reduction in total emergency operative figures, possibly due to more widespread use of non-operative strategies, there was still an increase in emergency inguinal hernia repairs during the lockdown. Further studies are needed to evaluate if the delays to elective operating will result in a long-term increase in the rates of emergency presentation.

## Introduction

Hernia repair is one of the most commonly performed elective operations [1]. When the coronavirus disease 2019 (COVID-19) pandemic started in the Spring of 2020, most European countries temporarily stopped elective hernia repair operations or kept these to a minimum [2]. Understandably, with the speed of onset of the pandemic, the uncertainties around the spread of the virus and its effects on health care workers and patients alike, multiple recommendations and often varying guidelines were issued by countries and medical organisations [3].

The Board members of the European Hernia Society (EHS) assisted by the senior author of this paper (BE) published guidance around hernia surgery in the current pandemic and looked at exit strategies as the pandemic diminished [4]. A conservative management approach to most hernias was seen as the safest option for many in the early months of the pandemic. For those presenting as an emergency, techniques like manual reduction under analgesia or sedation were also advised, successfully allowing safe early discharge of the patient from the hospital [5]. The aim of this study was to evaluate the impact of COVID-19 on the pattern of elective and emergency hernia surgery undertaken in the Lothian region of Scotland.

## Materials and methods

This is a retrospective cohort analysis. The local surgical database (Lothian Surgical Audit - LSA) was searched for all adult hernia operations performed between the beginning of the lockdown in Scotland on 23 March 2020 until the end of September 2020 [6]. Hernia repairs undertaken during the same time period in 2019 were also retrieved. A further local patient database (TRAK) was then used to determine the age and gender of the patients, the hernia location, the hernia repair technique (open or laparoscopic), and the urgency of the operation (elective or emergency). These data were collated and compared between the two study periods for differences in patient demographics, overall changes in the incidence of different techniques and urgency for total operations, and with further subgroup analyses by hernia location. The total hernia operations performed per whole month, with division into emergency and elective, were also drawn from the collated data to enable comparison over time within each study period. Statistical analysis was performed using the chi-squared test in R-Studio, with a p-value of <0.05 accepted as statistically significant [7].

## Results

A total of 570 hernia repair operations in 534 patients were undertaken in 2019. For the same time period, during 2020, there were 149 hernia operations in 142 patients. The mean age for all patients was 59.1 years in 2019 (range 19-94), and 57.7 years in 2020 (range 19-92).

The total number for each hernia type and the urgency of surgery (elective or emergency) are given in Table 1 below. Both the elective and emergency operation totals reduced from one study period to the next. The elective to emergency ratio was 5.95:1 (488 elective vs 82 emergency operations) for 2019 and 1.4:1 (87 vs 62) for 2020. This equated to a 27.2% reduction in the proportion of hernia repairs performed electively, from 85.6% elective in 2019 to 58.4% in 2020 (p<0.001).

**Table 1:** Hernia surgery case breakdown by clinical urgency % = percentage

Type	2019				2020			
	Operation Count	Elective	Emergency	% Emergency	Operation Count	Elective	Emergency	% Emergency
Inguinal	270	249	21	7.8	84	58	26	30.8
Umbilical	139	111	28	20.1	35	18	17	48.6
Incisional	82	74	8	9.8	6	2	4	66.7
Femoral	29	14	15	51.7	10	3	7	70.0
Epigastric	29	23	6	20.7	7	3	4	57.1
Spigelian	11	10	1	9.1	2	1	1	50.0
Parastomal	7	5	2	28.6	5	2	3	60.0
Obturator	2	1	1	50.0	0	0	0	0.0
Divarification	1	1	0	0.0	0	0	0	0.0
Total	570	488	82	14.4	149	87	62	41.6

On analysis of the composition of all operations by type subgroup, incisional hernia repair saw a significant reduction (p<0.001) in the proportion performed versus other types from 2019, with just six such procedures performed in 2020 (4% of all operations) as compared to 82 for the same period in 2019 (14% of all operations). In contrast, proportionally more inguinal hernias were repaired, making up 56% of all repairs in 2020, a rise of 9% in 2019 (p=0.0502), although total numbers decreased from 270 to 84 operations. No other hernia types were repaired at significantly different rates overall across the two study periods (p>0.05), although when measured by the absolute number of operations performed, large decreases were observed for all hernia types, as shown in Table 1.

The inguinal hernia subgroup had a 24% rise in emergency operations from 21 to 26 procedures, despite a reduction from 270 to 84 total inguinal repairs. In combination with the overall reduction in the operative count, this equated to a significant increase in emergency inguinal hernia repairs against all operations, from 4% of all operations in 2019 to 17% in 2020 (p<0.001). No absolute increases in emergency operations were observed in the other hernia type subgroups.

**Figure 1:**
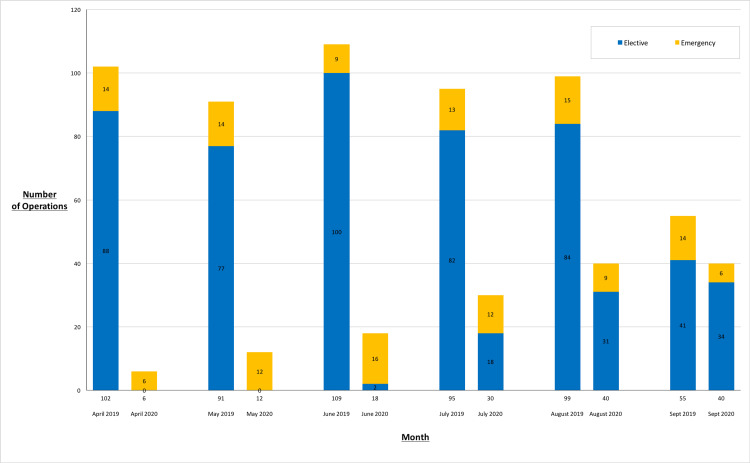
Number of hernia operations by whole month, 2019 vs 2020

There were just two elective hernia repairs carried out in the first three months of the 2020 study period (5.6% of all operations for April-June) compared to 265 (87.7%) for the same period in 2019 (p<0.001). The number of operations per whole month was relatively constant during April-August 2019, whereas it steadily rose throughout the same time frame the following year, starting from a total of only six operations in April 2020 (see Figure 1).

No statistically significant differences were observed in the rates of laparoscopic versus open operating techniques across the two study periods on any analysis, as shown in Table 2. This includes total operations (1% rise in laparoscopic, p=0.809) and various subgroup analyses: hernia types (Table 2), elective surgery (7% increase in laparoscopic, p=0.126), and emergency operations (4% rise, p=0.233).

**Table 2:** Hernia surgery case breakdown by operative technique % Lap = percentage of operations performed laparoscopically A p-value <0.05 was accepted as significant

Type	2019				2020				p-value
	Operation Count	Open	Laparoscopic	% Lap	Operation Count	Open	Laparoscopic	% Lap	
Inguinal	270	204	66	24.2	84	62	22	26.2	0.746
Umbilical	139	125	14	10.1	35	31	4	11.4	0.814
Incisional	82	73	9	11.0	6	6	0	0.0	-
Femoral	29	22	7	24.1	10	8	2	20.0	0.789
Epigastric	29	23	6	20.7	7	6	1	14.3	0.701
Spigelian	11	8	3	27.2	2	2	0	0.0	-
Parastomal	7	7	0	0.0	5	5	0	0.0	-
Obturator	2	1	1	50.0	0	0	0	0.0	-
Divarification	1	1	0	0.0	0	0	0	0.0	-
Total	570	464	106	18.6	149	120	29	19.5	0.809

The majority of hernia repairs were performed in men although the sex difference was less obvious when considering emergency patients only (Table 3). There was no significant difference in the male-to-female ratio of total repairs performed across the two study periods, 72.8% versus 67.8% male (p=0.209).

**Table 3:** Hernia surgery case breakdown by sex n = number % = percentage

	2019		2020	
Sex	Female, n (%)	Male, n (%)	Female, n (%)	Male, n (%)
All cases	154 (27.2)	416 (72.8)	48 (32.2)	101 (67.8)
Elective	116 (23.8)	372 (75.2)	21 (24.1)	66 (75.9)
Emergency	38 (46.3)	44 (53.7)	27 (43.6)	35 (65.4)

The average age for elective hernia repair in 2019 was 58.5 years (range 19-92), and it was 59.2 (range 19-89) in 2020. For emergency hernia repair, the average age for 2019 was 62.6 years (range 26-94), whilst in 2020, it was 55.5 (range 19-92).

## Discussion

This study has demonstrated that the volume of elective hernia surgery was markedly reduced in the six months following the onset of lockdown in the Lothian Region of Scotland, with the proportion of elective operations decreasing significantly from 85.6% to 58.4% (p<0.001). Elective operating in 2020 was just 18% of the 2019 total, with the reduction most marked in the first three months following lockdown, and the greatest impact seen on elective incisional hernia surgery, as noted in Table 1. Whilst there was a reduction in the number of emergency hernia operations in the six months following lockdown in 2020, this was not as marked as elective surgery (82% elective vs 24% emergency operation reduction from 2019 to 2020). The age and gender of the patients were similar over the two time periods, suggesting that there was no obvious discrimination in case selection against the elderly during the COVID-19 study period.

Reports from other countries have identified similar trends, although the absolute percentage reduction in elective hernia surgery has varied, partly due to the differing time periods of study and different working practices. Herniamed data showed a reduction in elective hernia surgery to 25% of usual [8] while Motol University Hospital reported a reduction of 67% from the normal [9]. Similar findings have been an absolute reduction in emergency hernia surgery, although to a much lesser degree than elective surgery.

A notable exception is that of the Greek experience shared by Mulita et al., who found the overall number of emergency operations to be unaffected (perhaps due to differing local experiences of COVID-19 at the time), and additionally observed a significant increase in the number of strangulated or irreducible inguinal and incisional hernias seen, as well as increased hospital stay and operation time for those emergency presentations [10,11]. This indicates the average severity of presentation at this centre was worse during the COVID-19 era, which may, unfortunately, be a trend we experience in the future.

Despite the overall reduction in emergency surgery we experienced, possibly due to more widespread non-operative interventions [5], inguinal hernia repairs still saw a 24% rise from 21 to 26 emergency operations during the lockdown in our study (Table 1). This, in particular, is a cause for concern, given that emergency hernia surgery is recognised to carry an increased risk of morbidity and mortality [1,12]. Whilst this increase is not completely unexpected, inguinal hernias are generally thought to be at low risk for complications [13].

Current guidance for groin hernias advises watchful waiting can be appropriate in the asymptomatic population, supported by evidence of a low complication risk, the expectation that symptoms developing will trigger a change in management, and a not-insignificant risk of post-operative chronic pain [1]. In contrast, given a lack of evidence for complication risk (strangulation and incarceration) in symptomatic patients, the guidance advocates elective surgical intervention [1]. Whilst delaying elective hernia surgery at the height of the pandemic was unilaterally agreed to be the safest option, this was expected to be for months rather than years [4]. Since then, the UK has come out of lockdown only to re-enter in the winter of 2020-21. This data shows the scale of the delays to elective surgery during the lockdown, and how this evolves in terms of emergency presentation and complication rates long-term remains to be seen.

There were no significant changes in any analysis for femoral hernia repair. It is well-established that femoral hernias tend to present more frequently as emergencies than other types, and indeed in our data, 15 of 29 (52%) were emergency operations prior to the pandemic, which could explain this finding.

Whilst the total number of emergency hernia operations also reduced, it is possible that this was because of increased non-operative therapies undertaken in the early period of the nationwide lockdown. Such a reduction in emergency hernia surgery has also been noted in other countries [14]. Nevertheless, it is likely that for many of those whose operations were cancelled or remain delayed, there is persisting impaired quality of life in a number of facets including the ability to work and play. As is the case for so many health care systems, including the Lothian Region of Scotland, the ability to catch up with the burden of hernia surgery care remains to be seen.

The proportion of elective laparoscopic hernia operations performed in our series increased from 21.3% in 2019 to 28.7% in 2020, but this was not significant (p=0.126). Similarly, there were no significant changes in operative practice noted on any laparoscopic subgroup analysis. Although there was debate regarding the safety of laparoscopic surgery during COVID-19, for fear of spreading infection with insufflation of body cavities [15], there was no consensus policy initially, and as the pandemic progressed evidence suggested this was not a significant concern [4].

Unfortunately, the COVID-19 infection rate for our 2020 cohort is not available, as inpatient testing was in its infancy at the beginning of the lockdown period. Regardless, it is likely not possible to determine if post-operative infections were truly a result of inpatient admission due to a combination of the incubation period of the virus, a high background rate of community infection at the time, and the fact that these operations often have short hospital stays. Whilst we, therefore, cannot comment from our data on the safety of undergoing surgery at that time, especially given there were just two elective hernia procedures in our region during the height of the pandemic (April-June 2020), the increased risks of post-operative complications with both active and resolved COVID-19 infection have been studied elsewhere [16], and additionally, the NHS infection prevention and control policies were deemed to be effective [17].

This is a study of the organisational challenges experienced by elective surgical services during a pandemic, rather than patient outcomes. However, a comparison of short-term outcomes across our two cohorts would have multiple confounding factors affecting the ability to draw meaningful conclusions; notably that they are not matched nor of similar size, that higher risk and frailer patients were more likely to have succumbed to COVID-19 infection and not present for surgery, as well as any intentional patient selection bias, and that rates of non-operative management are expected to be different between the two cohorts.

The main limitation of this study was that by its very nature, the total number of operations in 2020 is small, which impacted the ability to draw wider conclusions, particularly on subgroup analysis. Additionally, this study assessed the incidence of emergency presentation only by those that resulted in operative intervention. Lastly, this is an analysis of a single region and so the results may not be representative of other locations, particularly those with different patient demographics.

## Conclusions

The COVID-19 pandemic led to a marked reduction in the number of elective hernia repairs (especially incisional hernia surgery), with the effect most pronounced over the first three months of lockdown. Despite an overall reduction in total emergency operative figures, possibly due to the more widespread use of non-operative strategies, there was still an immediate increase in emergency inguinal hernia repairs during the lockdown. Further studies are needed to evaluate if the delays will result in a long-term increase in the rates of emergency presentation. Regardless, the elective waiting list backlog is expected to place a significant burden upon services for years to come as the country recovers, which may also impact surgical training.
